# Temporal dynamics of saccades explained by a self-paced process

**DOI:** 10.1038/s41598-017-00881-7

**Published:** 2017-04-20

**Authors:** Roy Amit, Dekel Abeles, Izhar Bar-Gad, Shlomit Yuval-Greenberg

**Affiliations:** 1 Sagol School of Neuroscience, Tel Aviv University, 6997801 Tel Aviv, Israel; 2 School of Psychological Sciences, Tel Aviv University, 6997801 Tel Aviv, Israel; 3 The Leslie and Susan Goldschmidt (Gonda) Multidisciplinary Brain Research Center, Bar Ilan University, Ramat Gan, 5290002 Israel

## Abstract

Sensory organs are thought to sample the environment rhythmically thereby providing periodic perceptual input. Whisking and sniffing are governed by oscillators which impose rhythms on the motor-control of sensory acquisition and consequently on sensory input. Saccadic eye movements are the main visual sampling mechanism in primates, and were suggested to constitute part of such a rhythmic exploration system. In this study we characterized saccadic rhythmicity, and examined whether it is consistent with autonomous oscillatory generator or with self-paced generation. Eye movements were tracked while observers were either free-viewing a movie or fixating a static stimulus. We inspected the temporal dynamics of exploratory and fixational saccades and quantified their first-order and high-order dependencies. Data were analyzed using methods derived from spike-train analysis, and tested against mathematical models and simulations. The findings show that saccade timings are explained by first-order dependencies, specifically by their refractory period. Saccade-timings are inconsistent with an autonomous pace-maker but are consistent with a “self-paced” generator, where each saccade is a link in a chain of neural processes that depend on the outcome of the saccade itself. We propose a mathematical model parsimoniously capturing various facets of saccade-timings, and suggest a possible neural mechanism producing the observed dynamics.

## Introduction

An increasing number of studies highlight the central role of rhythms in brain function, including attention and sensory sampling^1–4^. Some sensory organs sample the environment rhythmically thereby providing the neural sensory system with periodic input^5^. For example, whisking and sniffing, both predominant exploratory behaviors in rodents, are based on rhythmic motions of the facial muscles^6–9^.

Rhythmic behavior, such as whisking, could be generated either by the autonomous activity of a central oscillator, such as a “central pattern generator” (CPG); or by a self-paced loop driven by reafferent feedback from the movements themselves^10^. Previous studies showed that the generation and patterning of whisking movements are independent of sensory input as they persist even when it is absent. This indicated that whisking is generated by an autonomous CPG^10–13^.

In primates, saccadic eye movements are the main exploration mechanism by which the visual input is patterned into a series of 1–3 discrete samples per second^14, 15^. Observers produce saccades when scanning visual scenes, performing visual tasks^16^, and even during fixation^17, 18^. Most of these fixational saccades are very small and are termed “microsaccades”. Since saccades determine the visual inflow, explaining what governs their motor-control is vital for understanding the dynamics of vision. Despite this, not much is known about the temporal dynamics of saccades, and specifically, about the role of oscillations in saccade generation.

Previous studies showed that saccades timings are affected by prior saccades^19–23^ and by environmental factors, both bottom-up and top-down^16, 24, 25^. However, the abundant evidence on non-systematic environmental influences over saccades appears to be inconsistent with the observation that, similarly to other exploration behaviors, saccade sequences in humans show a rhythmic modulation at ~3–4 Hz^26^. The spectral properties of saccades are yet not well characterized, modelled and understood. Environmental and volitional influences are typically non-periodic and therefore cannot trivially explain this rhythmicity. The observed saccadic rhythmicity has led some to the speculate the existence of an oscillating generation process, possibly located in the motor cortex^27^, which was also thought to govern visual attention^26, 28–30^. According to this view, human saccades are modulated by the activity of autonomous oscillatory generators, similarly to exploration behaviors in rodents. These generators supposedly act in parallel to the non-periodic environmental influences, generating the observed saccadic rhythmicity.

The purpose of the present study was to explain saccadic rhythmicity. We tested the oscillatory-generation hypothesis against the alternative hypothesis that saccadic rhythmicity can be explained within the known framework of saccade generation, and without requiring an additional oscillatory process. Specifically, we examined whether the rhythmicity of saccades could be generated by the saccadic self-paced loop, driven by known properties of saccades: namely the saccadic refractory period^15, 20, 22^. Such generating model could be analogous to non-oscillatory spike generation models, where a Poisson process with a refractory period produces rhythmic spike-trains^31–33^. In such cases, where the timing of each spike in the series depends solely on the single previous event, the process is defined by its *first-order* intervals. Alternatively, when each spike depends on more than one preceding event, as in oscillatory spike-trains, the process is considered to be of a *high-order*. A process driven by autonomous activity of an oscillator would demonstrate high-order statistical dependencies.

Eye movements were tracked while observers were either free-viewing a movie or fixating a static stimulus. We inspected the temporal and spectral properties of exploratory and fixational saccades and quantified their first-order and high-order dependencies. Data were analyzed using methods derived from spike-train analysis, and tested against mathematical models and simulations. We examined the hypothesis that saccadic rhythmicity is driven solely by their first-order properties, and mainly by the saccadic refractory period, rather than by an autonomous high-order oscillation. According to this hypothesis, saccadic rhythmicity is “self-generated” as each saccade is a link in a chain of neural processes that depend on the outcome of the saccade itself. Based on the current findings combined with previous research, we propose a neural mechanism for the observed rhythmicity in saccade generation.

## Materials and Methods

### Subjects

Thirty-six students of Tel-Aviv University participated in three experiments for course credit or monetary compensation. All subjects reported normal (uncorrected) vision and no history of neurological disorders. The participants included 12 subjects (7 females; Mean age 25.9 ± 2.3) in Exp 1, 11 subjects (6 females; Mean age 26.6 ± 1.9) in Exp 2, with one additional subject rejected due to excessive blinking, and 12 subjects (7 females; Mean age 26.1 ± 2.7) in Exp 3. None of the subjects participated in more than one of the three experiments. All were naïve to the purpose of this study. The study was approved by the ethical committee of Tel Aviv University and that of the School of Psychological Sciences. All participants signed an informed consent. All experiments were performed in accordance with relevant guidelines and regulations.

### Stimuli

Exp 1: An 11 minutes long nature movie clip with sound. Exp. 2: A full contrast centered checkerboard (size 17° × 13°; spatial frequency 3 or 0.25 cycles/degree) presented on a mid-gray background. Exp. 3: Full contrast centered vertical or horizontal grating (size 17° × 13°, spatial frequency 3 cycles/degree), presented on a mid-gray background. In Exp 2–3 a 0.2° red fixation cross was displayed in the center.

### Procedure

Subjects sat, head resting on a headrest in a dimly-lit sound-attenuated chamber, at a distance of 57 cm from the display monitor. Eye-tracker calibration routine was applied at the beginning of each experimental session and repeated when needed. In Exp 1 a movie was presented and subjects were given no instruction other than to attentively watch it. In Exp 2–3 static stimuli were presented for the entire duration of each block (3 minutes) while subjects were instructed to fixate a central target. A tone was sounded to alert subjects when their gaze diverted for more than 1.5° away from the central fixation cross for more than 1 s.

### Eye tracking

Eye movements were monitored using a remote infrared video-oculographic system (Eyelink 1000 Plus; SR Research, Canada). Some doubts have been raised regarding the adequacy of video-oculographic eye trackers for measuring microsaccades^34^. However, their performance in detecting miniature fixational eye movements was found to be comparable to that of the invasive search coil technique, considered the gold-standard in this field^35^. When compared with other commercial devices, including the dual-purkinje-image (DPI) eye tracker, the Eyelink device was found to be among the highest in tracking precision^36^. Eye-gaze data were acquired using Eyelink software and offline filtered by a low-pass IIR Butterworth filter (cutoff 60 Hz; see Supplementary Figure 1). Saccades were detected using a published algorithm^37^. An elliptic threshold criterion for microsaccade detection was determined in 2D velocity space based on the horizontal and the vertical velocities of the eye-movement. Specifically, we set the threshold to be six times the SD of the eye-movement velocity, using a median-based estimate of the SD^37^. This threshold was set based on entire recording blocks (Exp 1: 11 minutes, Exp 2–3: 3 minutes). A saccade onset was detected only if six or more consecutive velocity samples were outside the ellipse, in both eyes. Saccades with peak velocity higher than 3 standard deviations from the mean in both eyes were assumed to be noise and removed from analysis (this resulted in the rejection of less than 0.1% of saccades).

Saccades offsets are sometimes accompanied by an “overshoot” which may be erroneously detected as a new saccade. Therefore, per standard procedure^38–40^ we imposed a minimum criterion (50 ms) for the interval between two saccade and kept only the first saccade if two saccades were detected in such proximity. The Saccade magnitude in visual degrees was calculated as the Euclidean distance from the starting position to the ending position of gaze. Saccades that were smaller than 1° were defined as microsaccades.

### Analysis

Analysis of the temporal properties of saccades was performed on “saccade sequences”: binary vectors representing all time samples in the experimental session, with ones for time points where a saccade onset was detected and zeros elsewhere.

#### Spectral analysis

Spectral analysis of saccade sequences was performed by calculating the average power of 10 s long windowed overlapping segments (Welch’s method), and then rescaling them to match the power as extracted from a single window^41^. The resulting spectral resolution is 0.1 Hz. We included only windowed segments in which the gaze data was constantly recorded (the eyes were not lost by the tracker) and there were no experiment breaks.

The postulated oscillatory modulation of saccade sequences was quantified using the Oscillatory-Modulation Index estimator $(\hat{m})$ ^41^. This measure, which provides an unbiased measure of the oscillation, is based on the estimated power of the modulation frequency Ŝ_f_, which was defined as the frequency with highest power in the range of 2–6 Hz.

1 $$\hat{m}=|\frac{2\cdot \sqrt{{\hat{{\rm{S}}}}_{f}-{r}_{0}}}{{r}_{0}\sqrt{L}}|$$

where L is recording length and r_0_ is the mean saccade rate.

Analysis of inter-saccade intervals (ISIs) was based on the time interval between all pairs of consecutive saccades. We then generated the time interval histogram (TIH) by fitting these intervals into 1 ms bins, and normalizing it to probabilities by dividing the bin count by total number of intervals. The TIH was smoothed using a 50 ms rectangular sliding window to create the probability distribution function (PDF).

#### Autocorrelograms

The autocorrelation function was calculated for each experimental- and model-based data-set using 1 ms bins, normalized to reflect saccade rate using standard methods^42^ and smoothed using a 50 ms rectangular sliding window. We defined a “flat autocorrelogram” as such where there was no peak greater than 2 standard deviations from the mean saccade-rate. A “peak autocorrelogram” was defined as such where there was a peak greater than 2 standard deviations from the mean. A peaked autocorrelogram was defined as oscillatory if the spectral power, in the time window starting after the initial peak, showed a peak greater than 2 standard deviations of its mean.

#### Hazard functions

The hazard function h(t) is the instantaneous probability for the occurrence of an event at time t (normalized TIH), divided by the probability that it has not occurred before (survival). It is defined as:

2 $$h(t)=\frac{P\{t\le T < t+dt|T\ge t\}}{dt},dt=0.001s$$

### Models

#### First-order model

A non-oscillatory model was designed to generate saccade sequences that matched the original data in saccade base-rate (r), recording length and modulation index $(\hat{m})$, but constituted of only first-order statistical dependencies–an inhibition period and a rebound after each event. Saccade occurrences were randomly determined based on a Poisson generator with a constant probability. For samples at post-saccade period, the probabilities were modulated to create an inhibition period and a post-saccade rebound in the following way:

3 $$p(t)=\{\begin{array}{ll}0 & {t}_{0} < t < {T}_{I}\\ (r\times b)/sr & {T}_{I} < t < {T}_{reb}\\ r/sr & t > {T}_{reb}\end{array}$$

where t_0_ is the preceding saccade time, r is the saccade base-rate of the original data-set, and sr is the sampling rate of the recording (1000 Hz). The duration of the inhibition period *T*_*I*_ was sampled from a Gaussian distribution function with a mean *μ*_*I*_ and standard deviation *σ*_*I*_. The post-saccade probability-rebound of magnitude b lasted for a constant *T*_*reb*_ time samples (*T*_*reb*_ > *T*_*I*_).

The parameters *μ*_*I*_ *σ*_*I*_ *T*_*reb*_ and b were fitted to original data by a systematic search through parameter space. The tested parameters were: 50 ≤ *μ*_*I*_ ≤ 500 with intervals of 20; 10 ≤ *σ*_*I*_ ≤ 100, with intervals of 10; 100 ≤ *t*_*reb*_ ≤ 500 with intervals of 20; 1 ≤ *b* ≤ 5 with intervals of 1. Goodness-of-fit (GOF) for each parameter set was calculated by a chi-square test between the resulting model’s ISI distribution and the original one. Using a standard procedure^43^, ISIs between 0 and 1000 ms were divided into k equiprobable bins. k was chosen to be the integer nearest to 1.88 × *n* ^2/5^ where n is the number of samples in the original ISI distribution^43^. The parameter set resulting in lowest Chi-square statistic was chosen for each data-set.

#### Oscillatory model

An oscillatory model was matched individually for each data set to create a simulated oscillatory saccade sequence of the same duration. The model was designed to generate a saccade sequence that matched the original data in saccade base rate (r), recording length (L), modulation index $(\hat{m})$ and modulation frequency (f_0_). This model was based on a similar random generator, this time modulated by a sinusoid function:

4 $$p(t)=(r\times \hat{m})\sin (2\pi {f}_{0}\times t)+r$$

Parameters $\hat{m}$, f_0_, r were matched to those of each individual data-set.

## Results

We performed three experiments with a total of 35 participants. We started with a free-view procedure which induced mostly large saccades (Exp 1; 8% microsaccades <1°). For comparability with previous studies on microsaccades we added a fixation procedure, which induced mainly small saccades (Exp 2; 88% microsaccades), and showed similar results. Since the background stimulation was chosen arbitrarily in Experiment 2 we replicated its findings with a third experiment with another background stimulation (Exp 3; 94% microsaccades). Consistently with previous findings^15^ we find shorter ISIs for free-view relative to prolonged fixation (*t*(34) = 2.14, *p* = 0.039). In the following, we describe all findings of the three experiments together, and then finish with a section comparing the results of the three experiments.

### Validity of detected saccades

To ensure that the detected saccades are not a consequence of eye tracking noise, we examined the main saccadic sequence^44^ and found that detected saccades of all three experiments followed the expected correlation between saccade velocity and amplitude. The r Pearson coefficient was >0.78 for all participants and experimental sessions. A one-way ANOVA showed that saccade rates varied between the three experiments (F(3, 44) = 5.04, p < 0.005, normality tested using Kolmogorov-Smirnov test p > 0.77 for all groups), and follow-up planned contrasts showed that this difference was due to higher saccade-rate during free-view (Exp 1: *Mean* = 1.74 ± 0.37 saccades/second; Values represent Mean ± SD) than during sustained fixation (Exp 2: *Mean* = 1.67 ± 0.74 saccades/second; Exp 3: *Mean* = 1.29 ± 0.24 saccades/second).

### Spectral properties of saccades

**Figure 1:**
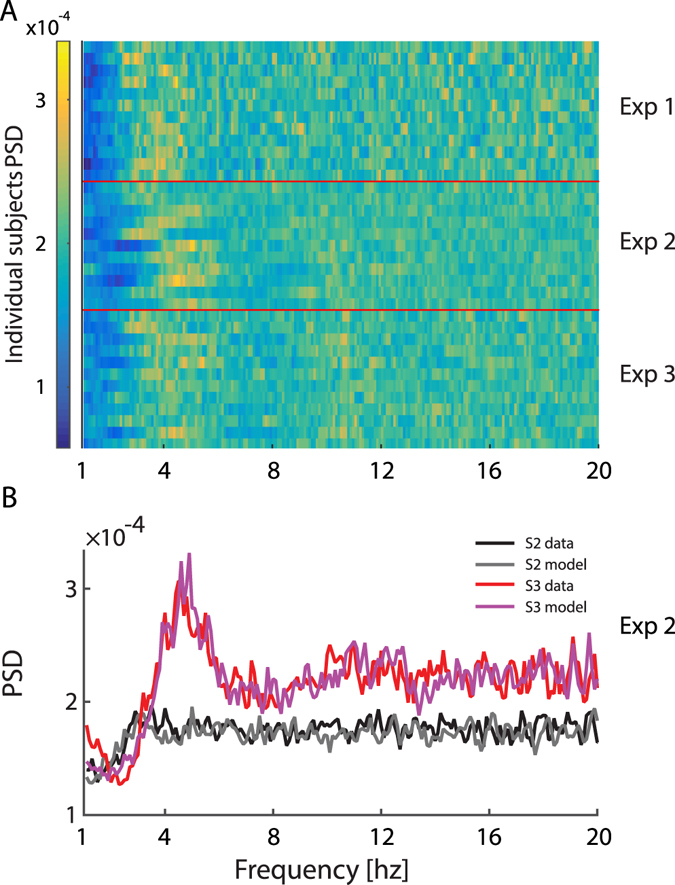
Spectra of saccade sequences. (**A**) Power spectra of saccade sequences. Each row represents the 1–20 Hz normalized power spectrum density of an individual observer. For convenience of presentation, we normalized each data-set’s power spectrum to the fraction of the overall power. (**B**) Same data for two selected observers. While one (S3-red line) has a distinctive spectral peak, the other’s spectrum is nearly flat (S2-black line). With different parameters, our first-order model can fit both types of data-sets (purple and gray lines).

We examined the power spectrum densities (PSD) in the range of 2–20 Hz. In line with previous accounts, saccade sequences were found to peak between 3–6 Hz for 23/35 (65%) observers (Fig. 1). At a first glance, this spectral peak could seem to contradict the saccade rate which is around 1–2 times per second (see previous paragraph). However, this apparent discrepancy is the result of the distribution of the inter-saccadic-intervals which had a wide exponentially decaying tail^15, 20^. Whereas the peak of the probability for the next saccade is around 200–300 ms, resulting in a spectral peak of ~4 Hz, the wide tail indicates that many saccades come at much longer latencies, thereby producing a much lower saccade rate than 4 per second.

For all experiments we found that the modulation index was close to 0.35 (Exp 1: *Mean* $\hat{m}$ = 0.37 ± 0.04; Exp 2: $\hat{m}$ = 0.36 ± 0.09; Exp 3: $\hat{m}$ = 0.36 ± 0.06) with mean peak frequency of 4 Hz (Exp1: *Mean* Ŝ_f_ = 3.77 ± 0.19 Hz; Exp 2: *Mean* Ŝ_f_ = 4.27 ± 0.21; Exp 3: *Mean* Ŝ_f_ = 4.1 ± 0.22) when searching the 2–6 Hz range.

### Inter-saccadic-intervals (ISI) probability distributions

**Figure 2:**
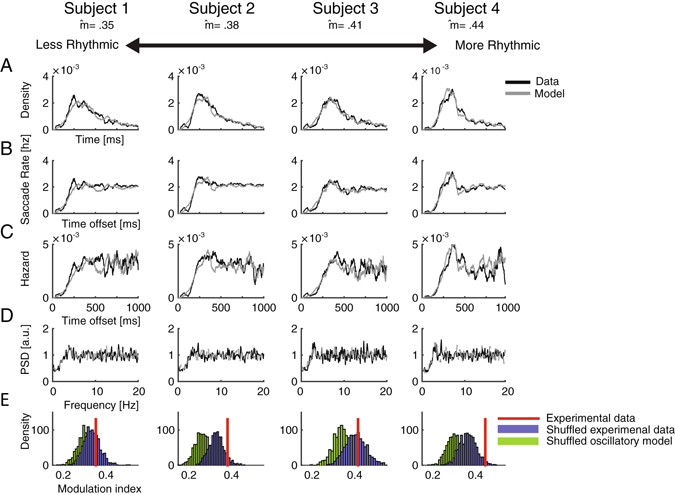
Statistical properties of single-subject saccade sequences during free-view (Exp 1). (**A–E**) Subjects ordered from left to right according to their level of rhythmicity as indicated by their modulation index. (**A**) Inter-saccade intervals distributions of 4 subjects (black) and their fitted first-order model (gray). (**B**) Autocorrelation functions of saccade events normalized to represent post-saccadic saccade rate. (**C**) Hazard Functions. (**D**) Power spectra of saccade sequences. (**E**) Histograms of modulation indices calculated on 1000 shuffles of intervals of either real data (blue) or simulated oscillatory data (green). The red bar indicates the original modulation index.

**Figure 3:**
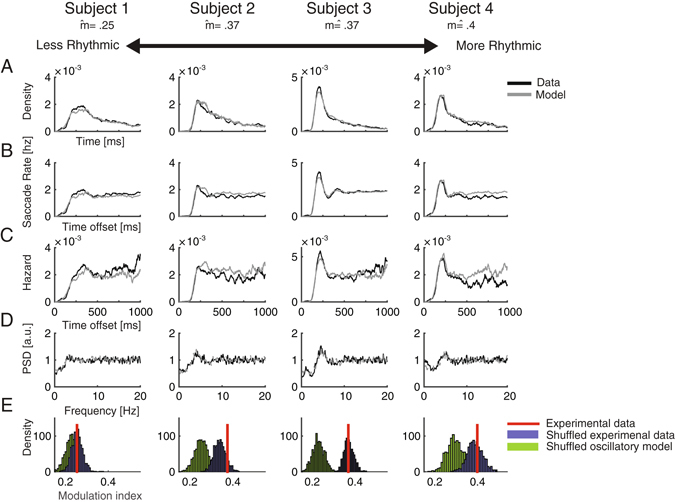
Statistical properties of single-subject saccade sequences during fixation (Exp 2). (**A–E**) Subjects ordered from left to right according to their level of rhythmicity as indicated by their modulation index. (**A**) Inter-saccade intervals distributions of 4 subjects (black) and their fitted first-order model (gray). (**B**) Autocorrelation functions of saccade events normalized to represent post-saccadic saccade rate. (**C**) Hazard Functions. (**D**) Power spectra of saccade sequences. (**E**) Histograms of modulation indices calculated on 1000 shuffles of intervals of either experimental data (blue) or simulated oscillatory data (green). The red bar indicates the original modulation index.

In line with previous findings^15, 20, 22, 23, 45^, the inter-saccadic-intervals (ISI) distribution of all data-sets showed a short period of inhibition, lasting 100–150 ms after the onset of each saccade, when no new saccades are generated (post-saccadic inhibition; Figs 2A and 3A). This is compatible with previous accounts of the post-saccadic “refractory period”^20–22^. This inhibition period is sometimes followed by a short rebound at 200–350 ms before returning to baseline rate. This phenomenon resembles the refractory period and its following probability burst found in neural firing^33^. Similarly to the refractory period and burst in neural firing, the inhibition and rebound are first-order dependencies, i.e. the probability of each event is affected solely by the previous one.

### Autocorrelations

The statistical interdependence of discrete events was thoroughly studied in the field of neural spike analysis. Saccades are statistically similar to spikes as they are stochastic discrete events which may be described as a point process. Therefore, it is beneficial to adopt some of the concepts of spike-train analysis when studying saccades^29^.

Three types of autocorrelograms were described in the context of spike-train analysis: *Flat autocorrelograms* reflecting neurons firing stochastically with constant probability for firing; *Oscillatory autocorrelogram* reflecting oscillatory neurons; and *Peak autocorrelogram* with a single short peak sometimes followed by a damped oscillation^31, 33^. The quantitative definitions of these terms are provided in the Methods section. The last type of autocorrelation is frequently observed for neural spikes and does not reflect the existence of a base-rate oscillation. Specifically, such peaked autocorrelograms were produced by stochastic processes which featured either an exceptionally high firing rate or a substantially long refractory period^46^. These peaked autocorrelograms may demonstrate a damped oscillation but they do not imply an underlying firing rate oscillation. For details on the categorization and quantification see Materials and Methods.

**Figure 4:**
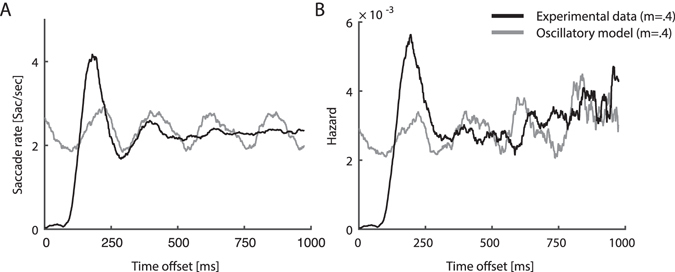
Comparison of saccade data and an oscillatory simulation model. (**A**) Autocorrelation function of subject 3 who participated in Exp. 2, normalized to represent post-saccadic saccade rate in Hz (black). The autocorrelation function of an oscillatory model matched in modulation index (gray). (**B**) Hazard function of the same data-set (black) and of its matched oscillatory model (gray).

None of our data-sets produced an oscillatory autocorrelation. In the free-view experiment (Exp 1), most autocorrelograms were flat (e.g. Fig. 2B Subjects 1–3, 10/12 - 83.3% of the observers) and only two were “peak autocorrelograms” (e.g. Fig. 2B Subject 4), resembling those shown for non-oscillatory neural spike-trains^33^. In the fixation experiments most autocorrelograms were “peak” (e.g. Fig. 3B subjects 3–4; 8/11 72% in Exp 2 and 8/12 66% in Exp 3) and the rest were “flat” (e.g. subjects 1–2 in Fig. 3B). For the peak autocorrelograms, the initial peak was followed by a short damped oscillation in few of the datasets (e.g. subjects 3–4 in Fig. 3B). None of the autocorrelograms in all experiments showed evidence for more than 1.5 rhythmic cycles (see the most periodic autocorrelations in Fig. 3B subjects 3–4). These findings indicate that the observed rhythmicity of saccade sequences is short-lived. In contrast, in an oscillatory simulation model matched to the data in modulation index, saccade rate and recording length, highly oscillatory autocorrelograms were obtained even with a weak oscillatory modulation (e.g. Fig. 4A).

### Hazard functions

The hazard function is the instantaneous probability of the occurrence of an event at time t from the previous event, given that it has not occurred before. Events that are driven by oscillations feature a periodic hazard function (Fig. 4B). In contrast, first-order statistical dependencies such as a burst and a refractory period in neural spikes may appear as a damped oscillation in the autocorrelation functions, but they do not produce a periodic modulation of the hazard function (Fig. 4B). For all experiments, the hazard functions of the saccade sequences showed only first-order dependencies and no evidence for a periodic modulation (Figs 2C and 3C). All hazard functions showed an initial dip in probability and this was followed for some by a rebound around 200–400 ms. Most hazard functions of the free-view experiment were flat with no rebound (e.g. Fig. 2C subjects 1–2; 10/12 - 83.3% of the observers of Exp 1). The rebound was found in approximately half of the data sets of the fixation experiments (e.g. Fig. 3C subjects 3–4; Exp 2: 6/11 54%; Exp 3: 6/12 50%). None of the hazard functions of the three experiments showed a periodic modulation, including those obtained from the seemingly more rhythmic data-sets (Fig. 3C subjects 3–4). This indicates that the observed rhythmicity originated solely from first-order and not from higher-order dependencies.

### Computational model

The first-order temporal structure of saccade sequences was verified by comparing them to a model without any high-order dependencies. A Poisson process constrained by a Gaussian inhibition period and a follow-up rebound was applied in a simple mathematical model (“first-order model”) which was implemented to simulate saccade data (see Materials and Methods). This model was fitted for each observer in terms of saccade rate and recording duration. Free parameters were optimized to produce ISI distributions which showed a good fit to the experimental data (Exp 1: *Mean χ* ^2^ = 0.06, *SD* = 0.03; Exp 2: *Mean χ* ^2^ = 0.05, *SD* = 0.02 Exp 3: *Mean χ* ^2^ = 0.06, *SD* = 0.03; Figs 2A and 3A). All data-sets showed a good fit to the model (all *χ* ^2^ < 0.12). Consistently with finding of flat hazard functions and autocorrelograms, in the free-view experiment (Exp 1) most (10/12, 83%) of the subjects showed a good fit to the model (*χ* ^2^ < 0.08) even with no burst modelled. This indicates that in the natural free-view task, most of the apparent rhythmicity can be parsimoniously accounted for with only a post-saccadic inhibition.

In contrast, in the fixation experiments, more data-sets required adding a burst for good fit (Exp 2: 8/11 72%, Exp 3: 4/12 33%). The more pronounced burst in the fixation conditions is visible when comparing Figs 2C and 3C.

Similarly to the experimental data, the output of the model was a peaked autocorrelogram, with a damped oscillation in some cases, depending on the specific parameters (Figs 2B and 3B). Despite having only first-order dependency features, this model captures all features that could be mistakenly considered to reflect the oscillatory nature of saccades: namely, their spectral properties (Figs 1B, 2D and 3D), the ISI distribution shape (Figs 2A and 3A) and the damped oscillation of some of the autocorrelograms (Fig. 3C).

**Figure 5:**
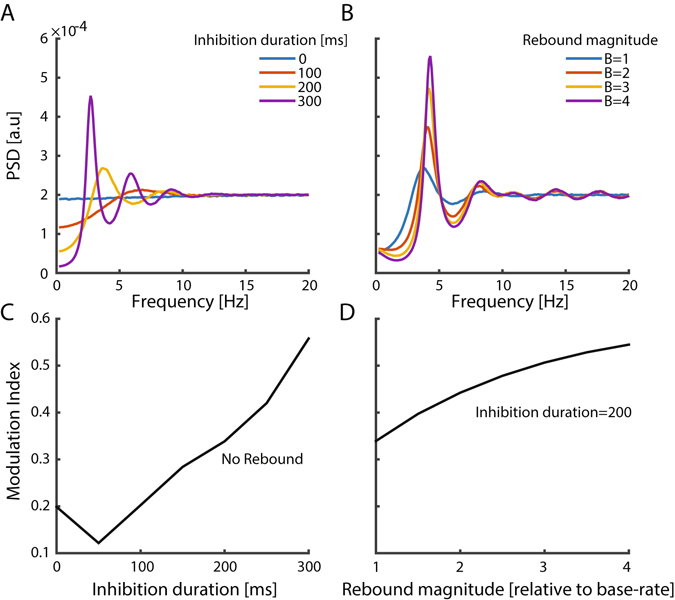
Effects of first-order model parameters on spectra and modulation indices. PSD (**A**), and modulation index (**C**), modulated by the duration of inhibition (rebound is fixed at 0). PSD (**B**) and modulation index (**D**) modulated by the magnitude of rebound (duration of inhibition is fixed at 200 ms).

To test the relative contributions of the two first-order dependency features (inhibition duration and rebound magnitude) to saccadic rhythmicity, we examined the performance of a simulation while varying the model’s parameters. The saccades spectral peak and the modulation index were positively correlated with the duration of the inhibition period (Fig. 5A,C) and the magnitude of the rebound (Fig. 4B,D), indicating that both parameters contribute to saccadic rhythmicity. Moreover, the inhibition period was found to be sufficient for inducing saccadic rhythmicity, as evident by a robust spectral peak produced by the simulation even when there was only a period of inhibition and no rebound (Fig. 5A). This analysis showed that the two first-order features have a dramatic impact on the rhythmicity of the saccadic process, in line with previous work^32^.

### Statistical evaluation of high-order dependencies in saccade sequences

**Figure 6:**
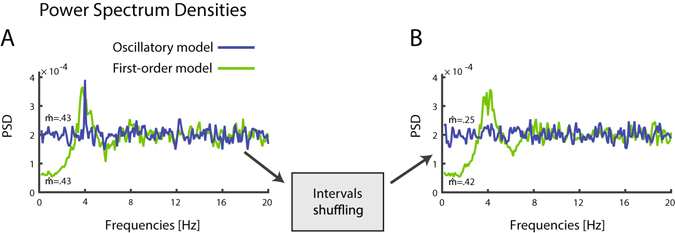
Modulation of the power spectra by shuffling. (**A**) Spectra of oscillatory model and first-order model matched in spectral peak magnitude. (**B**) After shuffling, the spectrum of the oscillatory model is flattened while the spectrum of the first-order model is preserved.

The hypothesis that saccade-sequences contain only first-order and no higher-order statistical dependencies was assessed by showing that disrupting high-order statistics has only negligible effect on the experimental data, compared to its effect on simulated oscillatory data. Disruption of high-order dependencies was achieved by shuffling the ISIs to form new sequences equivalent to the original in every respect except that they had no high-order dependencies. This shuffling procedure preserves the dependency between each saccades and a single preceding saccade, while obliterating dependencies with earlier saccades in the sequence. Therefore, following shuffling the spectral properties of a first-order process are preserved, while the spectral peak of an oscillatory process is diminished (Fig. 6A,B).

### Shuffling does not affect the modulation index of saccades sequences

**Figure 7:**
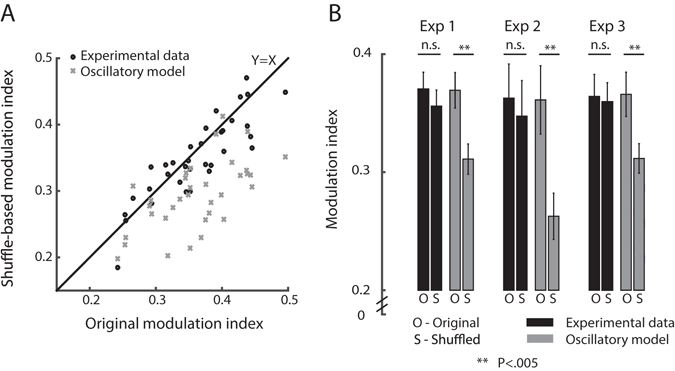
Modulation indices of individual subjects and groups. (**A**) For each subject, the shuffled sequence modulation index is plotted as a function of experimental modulation index (black circles). As a baseline for this analysis, the shuffled simulated oscillatory sequence modulation index is plotted as a function of the original modulation index (gray Xs). (**B**) For each experiment, the mean modulation index before and after shuffling is given, both for experimental data (black) and oscillatory model (Gray). Data are represented as mean ± SEM, N = 12,11,12 for Exp 1–3 respectively.

We simulated saccade sequences from each experimental data-set by shuffling the ISIs and reconstructing a surrogate saccade sequence. We obtained a null distribution for non-oscillatory modulation index by repeating this surrogate data construction 1000 times, each time recalculating the modulation index. For each data-set, the proportion of the null distribution greater than the measured modulation index was designated as the one-tailed p-value. In 32/35 (91%) of the data-sets the modulation index was not significantly lowered by shuffling (one tailed and not corrected for multiple comparisons; p > 0.05) (Fig. 7A).

The same analysis was performed on simulated oscillatory data to validate that oscillatory data would be affected by this shuffling procedure. To do this, we constructed simulated oscillatory saccade sequences, which matched for each data-set in modulation index, recording duration and saccade-rate. Despite the rather weak oscillatory modulation of these simulations (determined by the low modulation indices of the experimental data-sets), the shuffling procedure showed significant decreases in modulation index in 18/35 (51%) of the data-sets (one tailed; p < 0.05). This analysis confirmed that there is a qualitative difference between shuffling oscillatory models and shuffling original saccade-sequences.

Theoretically, it could have been expected that all oscillatory models data-sets would show significant decrease in the modulation index following the shuffling procedure, and not only 51%. However, some of the included data-sets had low modulations indices, and therefore their matched oscillatory model also had only a minor oscillatory modulation. Indeed, the 49% of data-sets which showed no significant decrease in modulation index, had lower modulation indices (*Mean* $\hat{m}$ = 0.32) compared to the rest (*Mean* $\hat{m}$ = 0.41; t_32_ = 4.4, p < 0.0001), and were therefore less susceptible to contain an underlying oscillation to begin with.

### Shuffling affects the modulation index of the oscillatory data more than the original data

We tested whether the shuffling procedure affects the oscillatory data significantly more than it affected the original data (as illustrated in Figs 6 and 7). This was done by obtaining for each subject 1000 modulation indices from iteratively shuffling the experimental data, and 1000 modulation indices from iteratively shuffling the oscillatory simulated data. A randomization-test was then performed separately for each subject (see Materials and Methods) to validate the modulation indices of the shuffled simulated data are lower than those of the shuffled experimental data. The effect was found to be significant in 31/35 of the subjects (88.5%; Figs 2E and 3E).

We performed the same analysis at the group level. Averaged modulation indices were calculated for each subject, across the 1000 shuffling iterations on both the original data ($\hat{m}$_shuff-orig_) and the simulated oscillatory data ($\hat{m}$_shuff-osci_). The $\hat{m}$_shuff-osci_ were systematically lower than the $\hat{m}$_shuff-orig_ (Fig. 7), and this effect was significant across subjects (Exp1: p < 0.001; Exp 2: p < 0.001; Exp 3: p < 0.01; Independent two-sample permutation test).

In summary, these findings show that shuffling the ISIs of saccade sequences does not have a significant impact on their oscillatory nature. This is in contrast to the robust impact shuffling has on simulated oscillatory data, which has the same level of rhythmicity as the original data but is derived from an oscillatory process. This suggests that the original saccade sequences data features first-order and no high-order statistical dependencies, both in natural free-viewing conditions (Exp 1) and in fixation tasks (Exp 2–3).

### Comparing different viewing conditions and saccade sizes

The experimental procedures included both a prolonged fixation condition and free-viewing of a movie. The fixation condition was included because it presented the strongest version of our argument: that even small, involuntary saccades during fixation (i.e. microsaccades) in the absence of external visual changes are not modulated by an oscillation. The free-view condition was included to show that even with dynamic rapidly-changing stimulation, saccades show a similar ~4 Hz rhythm comparable to that observed during steady fixation.

The findings described above show no evidence for high-order dependencies both in free-view (Exp 1) and in fixation (Exp 2 and 3) and with two different visual stimulus types (Exp 2 and 3). Therefore, the main conclusion of the current study is applicable for both natural free-viewing conditions and laboratory-produced fixation task. However, there was a difference between the two viewing conditions (fixation vs. free-view) in the nature of the first-order dependencies between saccades. Specifically, most of the free-view datasets, but only a few of the fixation datasets, could be modeled without incorporating burst. We speculate this is due to the fact that a microsaccade occurring in a fixation task typically draws the gaze away from the target of fixation, and this may cause a fast corrective microsaccade back to fixation^47^. In contrast, during free-view there is no defined fixation target and therefore most saccades shift the gaze to new fixation targets with no urgent need to correct back to the original location. The modulation index did not vary between free-view and fixation (t_33_ = 0.028; p = 0.77). When comparing the parameters fitted by the first-order model, we found a significant difference only in the duration of the inhibition period, which was longer in the free-view experiment *μ*_*I*_ (t_33_ = 2.7 p = 0.01; FDR corrected for multiple comparisons^48^), and no significant differences for the rest of the parameters (*σ*_*I*_: t_33_ = 1.67 p = 0.1; *T*_*reb*_: t_33_ = 1.86 p = 0.07; and b: t_33_ = −0.3 p = 0.74; The critical pFDR for an alpha of 0.05 was 0.01).

The different backgrounds used in Exp 2 and 3 did not affect any of the examined features: including modulation index (t_21_ = 0.04, p = 0.96), modulation frequency (t_21_ = −0.50, p = 0.59), saccade rate (t_21_ = −1.71, p = 0.1), or any of the model parameters (*μ*_*I*_: t_21_ = −1.6237 p = 0.11 *σ*_*I*_: t_21_ = 0.49 p = 0.62; *T*_*reb*_: t_21_ = 0.82 p = 0.41; and b: t_21_ = 0.69 p = 0.49; uncorrected for multiple comparisons).

### Handling blinks

**Figure 8:**
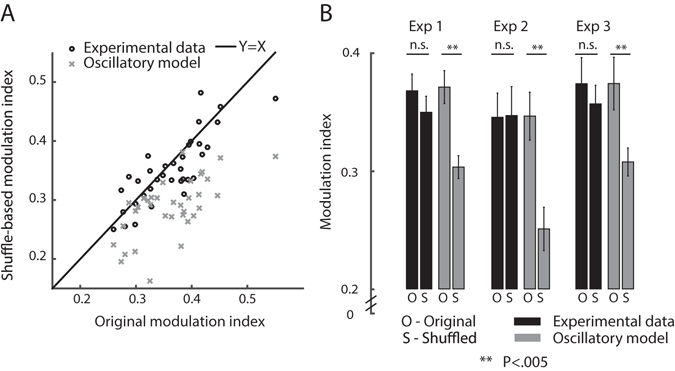
Modulation indices of individual subjects and groups (surrogate blink-free data). This figure is equivalent to Fig. 7 remade using blink-free saccade sequences. (**A**) For each subject, the shuffled sequence modulation index is plotted as a function of real modulation index (black circles). As a baseline for this analysis, the shuffled oscillatory sequence modulation index is plotted as a function of the original modulation index (gray Xs). (**B**) For each experiment, the mean modulation index before and after shuffling is given, both for real data (black) and oscillatory model (Gray). Data are represented as mean ± SEM, N = 12,11,12 for Exp 1–3 respectively.

Saccades are influenced by other oculomotor events, such as blinks. All the aforementioned analyses were performed on saccade sequences ignoring the occurrences of blinks, and consequently blinks should have not influence our conclusions. However, to avoid any lingering doubts regarding the effects of blinks, we reanalyzed the data after constructing tailored saccade-sequences which included no blinks. We did that by first marking only intervals without intervening blinks and then concatenating these intervals to form tailored continuous saccade-sequences with no blinks. We repeated all analyses on these saccade sequences and found qualitatively similar results: 1/35 of hazard functions and autocorrelations were oscillatory, most of autocorrelograms and hazard functions are flat (autocorrelograms: 57%; hazard: 60%). In 30/35 (85%) of the data-sets the modulation index was not significantly lowered by shuffling, in contrast to 48% of the oscillatory surrogate datasets. Figure 8 presents the results of this analysis, which are comparable to those shown by the similar Fig. 7 above.

## Discussion

In this study we characterized the temporal dynamics of saccades. The findings show that saccade rhythmicity can be parsimoniously explained by first-order statistical dependencies and are not modulated by high-order features. In agreement with a first-order generation model, the autocorrelation functions showed only a short-lived rhythmicity and the hazard functions demonstrated no periodicity. Furthermore, a simple first-order mathematical model fully accounted for various facets of the data. Finally, shuffling the intervals between saccades showed that rhythmicity is not disrupted even when only first-order and no high-order dependencies are preserved.

The main purpose of the saccadic system is to shift the gaze to points of interest in the visual environment^22, 49^. Previous studies showed that the dynamics of visual exploratory behavior are determined by the properties of the visual scene (bottom-up) and by top-down influences^16, 22^, including volitional control to allow goal-driven exploration^15, 50^. These influences of environmental and volitional factors could have led to the conclusion that saccades are governed solely by these factors. However, in what seems to be inconsistent with the central role of environmental factors, saccade-timings are rhythmically modulated. This rhythmic modulation cannot be trivially explained by environmental factors, which do not necessarily have a periodic influence, and has led some to suggest that there is a central oscillator modulating saccade generation^14, 26, 28–30^, along the influences of task and stimulation. The present findings provide an alternative parsimonious solution to the apparent contradiction between the rhythmicity of saccades and their modulation by environmental factors. The findings show that the complex saccadic behavior is constrained by simple first-order processes. These constraints, and mainly the previously-known saccadic refractory period^15, 20, 22^, are the core factors determining the rhythmicity of visual exploration.

A process showing only first- and no higher-order dependencies, can be explained by a self-paced generating mechanism, which is driven by its own occurrences. This is a parsimonious explanation as it requires no additional mechanism other than the observed one, which regenerates itself at each step. Our findings suggest that saccades can be explained by such a self-paced process: each saccade is a link in a chain of neural processes that depend on the outcome of the saccade itself, and are independent of any external pace-makers. The outcome of each saccade solely determines the timing of the following saccade. Notably, the present findings cannot exclude the involvement of additional mechanisms whose influence may have been too minor to be detected by the analysis. Given the simplicity and completeness of the proposed mechanism and the lack of contradicting evidence, the existence of an additional mechanism driving saccades is unlikely.

Some rhythmic neural processes are based on first-order mechanisms. For instance, excitatory-inhibitory feedback loops, which are known to induce periodic neural activity^51, 52^ have only first-order dependencies and could theoretically be driving the observed first-order saccades. However, our findings indicate that the outcome of saccades must be part of any loop governing their generation. An excitatory-inhibitory loop could theoretically be generating saccades, but this would be possible only if either the visual or the motor consequences of saccades modulate this loop. This means that when a saccade is, for instance, delayed, the timing of the next saccade would be determined not be an independent rhythmic process but by the previous delayed saccade. This self-paced generating mechanism makes saccades inherently different than other exploratory systems, including whisking and sniffing, which are governed by autonomous oscillatory drives.

We hypothesize that most of the first-order dependencies observed for saccades originate from two basic features: a) A Gaussian-distributed post-saccadic inhibition period: a period of time post saccade when a subsequent saccade is less likely to be generated; and for some observers also b) A rebound: a short-term increase of saccade probability at the end of the inhibition. The validity of this hypothesis is supported by earlier studies, which reported a saccadic inhibition period lasting for 150–200 ms following each saccade^20–22^.

The model we present simulates a stochastic process constrained by inhibition and rebound. This non-oscillatory model provides a good fit to the saccadic sequences data, supporting the role of inhibition and rebound in determining the dynamics of saccades. These first-order features can fully explain the saccadic rhythmicity, without requiring an additional base-oscillation.

Our design included a fixation and a free-view condition and therefore we examined mainly fixational saccades (mostly microsaccades) and explorative saccades (large and small). Saccades and microsaccades form an oculomotor continuum as they share the same kinematic properties and are controlled by the same neural structures^15, 53^. Consistently, saccades and microsaccades share the same basic statistical dependency properties, i.e. they are similarly driven by first-order dependencies. The difference found between the two conditions, i.e. longer saccadic inhibition and a less pronounced burst in free-view, are probably due to the different task demands and stimulation and not to differences in saccade sizes.

Recent evidence suggests that following a reset cue, behavioral performance fluctuates at theta frequency^54–56^. Theta rhythmic modulations of gamma-band activity, observed in the visual cortex were related to this attentional fluctuation^28^. The “communication through coherence” (CTC) model suggests that neural theta rhythm reflects the periodic termination of attentional engagement and shifts of attention from one attended target to another^3, 54, 57, 58^. This theta rhythm was suggested to drive both performance fluctuations and rhythmic sensory sampling behaviors, including saccades^26, 30^. Indeed, visual cortical oscillations at this frequency are highly synchronized with the occurrences of saccades^26, 28, 59–61^.

**Figure 9:**
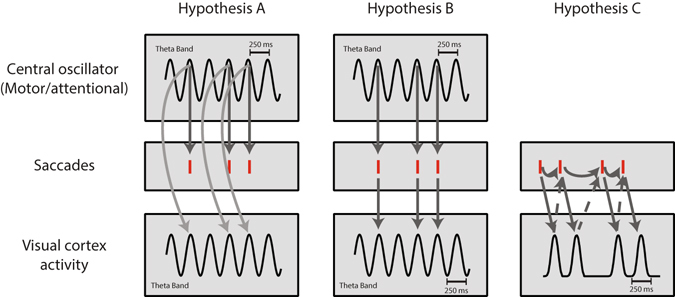
Alternative models explaining visual exploration dynamics. Hypothesis A Central theta oscillations drive saccades and the visual cortex independently; Hypothesis B, Central theta oscillations drive saccades. Saccades then drive visual activity through retinal image shifts or efferent activity; Hypothesis C, Saccades are generated by a self-paced mechanism and drive cortical rhythmicity through retinal image shifts or afferent activity. The visual transient caused by saccades’ first-order statistics constitutes the observed cortical theta rhythm (solid arrows).Visual cortex activity then drives saccades (dashed arrows).

A few different hypotheses could explain the relation between saccades and cortical oscillations. According to some, the fluctuations of a central oscillator drive the visual cortex either directly (as is depicted in Fig. 9, Hypothesis A) or indirectly by driving saccades which, in turn, modulate the visual cortex through retinal image shifts and afferent activity (Fig. 9, Hypothesis B). Our current findings show that rhythmic saccade sequences can be explained by a self-paced generating process (Fig. 9; Hypothesis C, dashed arrows). This view is inconsistent with the hypothesis that saccades are part of a general exploration system driven by cortical oscillations. Models suggesting that sensory exploration is driven by a synchronizing oscillator, which were validated in other sensory modalities, such as whisking and sniffing^9, 10, 62–64^, do not provide the most parsimonious explanation to account for the dynamics of the visual saccadic exploration system.

Evidence from EEG, fMRI and single-cell recordings show robust modulations of the visual cortex by single saccade onsets which are mostly due to the visual transients caused by retinal image shifts^59, 60, 65^. We hypothesize that the first-order dynamics of saccades, which induce a saccadic theta rhythm, translate into a modulation of visual activity at the same frequency^28^. We conclude that cortical oscillations, which correlate with saccades, are more likely *driven* by the saccades than *driving* them.

### The neural basis of self-paced saccadic exploration

Post-saccadic inhibition, which is the main first-order feature involved in driving saccadic exploration, could be either controlled by the visual cortex (dashed arrows, Fig. 9, Hypothesis C) or by subcortical oculomotor structures. A series of studies have investigated the neural source of another well-known and similar phenomenon called the “post-stimulus saccadic inhibition”^66–68^ or “the oculomotor freeze”^25^. Following the presentation of a stimulus, saccade rate drops and then increases in a rebound before returning back to baseline. Some researchers have speculated that post-stimulus saccadic inhibition is controlled by subcortical circuits such as the retinotectal pathway to the Superior Colliculus^17, 24, 69–71^, but a recent study^25^ showed that the saccadic inhibition following a stimulus is correlated with its conscious perception and therefore is most likely mediated by the visual cortex. According to this view, the stimulus signal is processed in the visual cortex and then transmitted into subcortical oculomotor control centers, where it triggers saccadic inhibition and release.

We speculate that saccadic inhibition following a saccade and saccadic inhibition following a stimulus are in fact the same phenomenon. It was previously shown that the visual transient triggered by saccadic retinal image shift produces the same neural effect as that caused by the presentation of a stimulus^72–74^. Consequently, the visual transient caused by a saccade produces the typical post-stimulus temporal dynamics of saccade-rate (inhibition followed by rebound), and that is the observed “post-saccadic inhibition”. If this hypothesis is true, the neural mechanism suggested to control the post-stimulus saccadic dynamics is the same one controlling the post-saccadic inhibition.

We propose a neural mechanism driving saccades, which is based on first-order features and comprises an alternative for an oscillatory generating mechanism. Each saccade triggers a visual transient, which activates the visual cortex through the Geniculostriate pathway. The visual cortex then relays feed-back signals to subcortical oculomotor centers, which produce a transient inhibition of saccades. Following the release from inhibition and onward, saccades may be triggered stochastically but are also modulated by top-down influences such as goal-directed exploration or bottom-up as a result of visual changes. As our model demonstrated, this inhibition (sometimes followed by rebound) is enough to create the observed statistical properties of saccadic sequences, including their rhythmicity, without requiring an oscillating generator.

### The sinusoidal oscillatory model

The baseline for the main analysis of this study was an oscillatory simulation model, which was designed by applying a sinusoidal modulation on a series of stochastic events. However, brain rhythms are typically not purely sinusoidal, but rather comprise of multiple harmonics. In a recent new version of the CTC Fries^3^ proposed that the rhythmic modulation of neural excitability is governed by an excitation-inhibition cycle which has a short excitation period followed by a longer inhibition period. This leads to a non-sinusoidal gain modulation.

By choosing of a sinusoidal modulation for our baseline simulation model, rather than an uneven duty cycle rhythm which includes significant power in harmonics of the base frequency, we took a conservative approach. By choosing the modulation index of the base frequency, which in our case was also significantly larger than all other harmonics, we tested the largest possible external modulation of the signal.

## Conclusion

To conclude, saccades are the main visual exploration mechanism. They provide repeated sampling of the visual environment and are the most prominent factor determining visual input. Therefore, the temporal dynamics of saccades dictate the temporal dynamics of the visual input flow, and that, in turn, dictates the temporal dynamics of visual cortical processing. Considering this, any model accounting for visual brain activity remains deficient unless the first-order dynamics of the source of input (i.e. saccades) are taken into account. In this study we characterized the temporal and spectral properties of saccades and proposed a parsimonious mechanism explaining their rhythmicity using their first-order characteristics. The rhythm of saccades can be explained by a self-paced generating loop including feedback connections from the visual cortex to subcortical areas, without requiring an oscillatory generator. According to our view, rather than being driven by cortical oscillations, saccades and their first-order properties may in fact be a critical source of rhythm injected into the neural system.

## Electronic supplementary material

Supplementary material
