# Supplementary material for: Temporal dynamics of saccades explained by a self-paced process
Source: Sci Rep. 2017 Apr 20;7:886. doi: 10.1038/s41598-017-00881-7 (PMC5430543; doi:10.1038/s41598-017-00881-7)

**Temporal dynamics of saccades explained by a self-paced process**

Roy Amit, Dekel Abeles, Izhar Bar-Gad and Shlomit Yuval-Greenberg

**Figre S1.** *Saccade detection using different lowpass filter cutoffs.* Left: Raw horizontal and vertical gaze position. Detected saccades following the application of different filters are marked by: red x’s (60Hz), green circles (100Hz) and light-blue circles (150Hz). Out of these three low-pass-filters, 60Hz gave the best detection results. Right: The left bar graph represents correlation between saccade velocity and amplitude (the main saccadic sequence) following the application of the three filters. The right bar graph represents the number of detected saccades following the same filters. Our of the three examine filters, 60Hz gave the best results, both in terms of a high velocity-amplitude correlation (indicating there are not many false alarms) and in term of the number of saccades (indicating that there are not many misses).


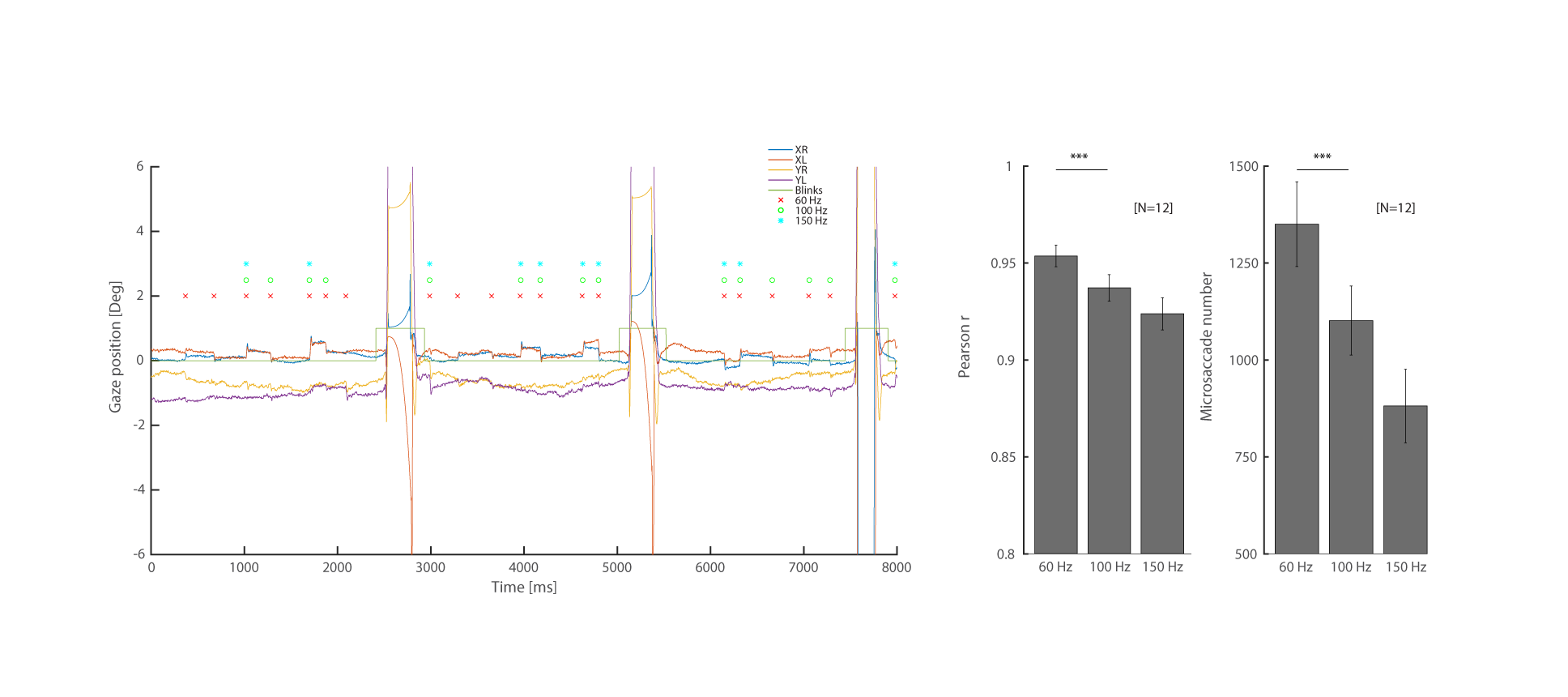

Supplement: Supplementary file 1 — Supplementary material [file 41598_2017_881_MOESM1_ESM.docx]
